# Machine Learning Models and Multiparametric Magnetic Resonance Imaging for the Prediction of Pathologic Response to Neoadjuvant Chemotherapy in Breast Cancer

**DOI:** 10.3390/cancers14143508

**Published:** 2022-07-19

**Authors:** Carmen Herrero Vicent, Xavier Tudela, Paula Moreno Ruiz, Víctor Pedralva, Ana Jiménez Pastor, Daniel Ahicart, Silvia Rubio Novella, Isabel Meneu, Ángela Montes Albuixech, Miguel Ángel Santamaria, María Fonfria, Almudena Fuster-Matanzo, Santiago Olmos Antón, Eduardo Martínez de Dueñas

**Affiliations:** 1 Medical Oncology Department, The Provincial Hospital of Castellon, 12002 Castellon, Spain; silruno@gmail.com (S.R.N.); angelamontes26@gmail.com (Á.M.A.); mariafonfria@gmail.com (M.F.); olmos.santiago@gmail.com (S.O.A.); eduardo.martinez@hospitalprovincial.es (E.M.d.D.); 2 Radiodiagnosis Department, The Provincial Hospital of Castellon, 12100 Castellon, Spain; xavier.tudela@hospitalprovincial.es (X.T.); victor.pedralva@hospitalprovincial.es (V.P.); dahisaf@me.com (D.A.); isabel.meneu@hospitalprovincial.es (I.M.); miguelangel.santamaria@hospitalprovincial.es (M.Á.S.); 3 Quantitative Imaging Biomarkers in Medicine (Quibim), 46021 Valencia, Spain; paulamoreno@quibim.com (P.M.R.); anajimenez@quibim.com (A.J.P.); almudenafuster@quibim.com (A.F.-M.)

**Keywords:** multiparametric MRI, radiomics, imaging biomarkers, machine learning

## Abstract

**Simple Summary:**

Achieving pathological complete response (pCR) to neoadjuvant chemotherapy (NAC) in breast cancer (BC) is crucial, as pCR is a surrogate marker for survival. However, only 10–30% of patients achieve it. It is therefore essential to develop tools that enable to accurately predict response. Recently, different studies have demonstrated the feasibility of applying machine learning approaches to non-invasively predict pCR before NAC administration from magnetic resonance imaging (MRI) data. Some of those models are based on radiomics, an emerging field that allows the automated extraction of clinically relevant information from radiologic images. However, the research is still at an early stage and further data are needed. Here, we determine whether the combination of imaging data (radiomic features and perfusion/diffusion imaging biomarkers) extracted from multiparametric MRIs and clinical variables can improve pCR prediction to NAC compared to models only including imaging or clinical data, potentially avoiding unnecessary treatment and delays to surgery.

**Abstract:**

Background: Most breast cancer (BC) patients fail to achieve pathological complete response (pCR) after neoadjuvant chemotherapy (NAC). The aim of this study was to evaluate whether imaging features (perfusion/diffusion imaging biomarkers + radiomic features) extracted from pre-treatment multiparametric (mp)MRIs were able to predict, alone or in combination with clinical data, pCR to NAC. Methods: Patients with stage II-III BC receiving NAC and undergoing breast mpMRI were retrospectively evaluated. Imaging features were extracted from mpMRIs performed before NAC. Three different machine learning models based on imaging features, clinical data or imaging features + clinical data were trained to predict pCR. Confusion matrices and performance metrics were obtained to assess model performance. Statistical analyses were conducted to evaluate differences between responders and non-responders. Results: Fifty-eight patients (median [range] age, 52 [45–58] years) were included, of whom 12 showed pCR. The combined model improved pCR prediction compared to clinical and imaging models, yielding 91.5% of accuracy with no false positive cases and only 17% false negative results. Changes in different parameters between responders and non-responders suggested a possible increase in vascularity and reduced tumour heterogeneity in patients with pCR, with the percentile 25th of time-to-peak (TTP), a classical perfusion parameter, being able to discriminate both groups in a 75% of the cases. Conclusions: A combination of mpMRI-derived imaging features and clinical variables was able to successfully predict pCR to NAC. Specific patient profiles according to tumour vascularity and heterogeneity might explain pCR differences, where TTP could emerge as a putative surrogate marker for pCR.

## 1. Introduction

Globally, breast cancer (BC) is the most commonly diagnosed cancer and the most common cause of cancer death among women [1]. In the management of BC, neoadjuvant chemotherapy (NAC) has been well established for patients with large breast tumours, high-risk breast tumours and locally advanced tumours, including those initially ineligible for surgery [2]. Although different NAC regimens exist, anthracyclines followed by taxanes are widely used chemotherapeutic agents, as they have been demonstrated to significantly increase the rate of pathological complete response (pCR) [3,4]. Additionally, targeted monoclonal antibody therapies against tumour biological properties—such as the expression of the human epidermal growth factor receptor 2 (HER2)—have progressively been incorporated into standard regimens, becoming an additional option that is generally well-tolerated [5,6].

NAC provides clear benefits to patients, including the reduction in tumour size, which facilitates surgical resectability, as well as the increase in breast-conserving surgery rates [7]. It also enables an objective evaluation of treatment efficacy, allowing therapy changes if desirable responses are not achieved [7]. Indeed, NAC has become a useful setting for the assessment of new anticancer therapies, with pCR arising as a surrogate endpoint for treatment efficacy [8]. Additionally, pCR has consistently been associated with improved clinical outcomes, including overall survival (OS), event-free survival (EFS) and disease-free survival (DFS), at least when pCR is defined as ypT0 ypN0 (absence of invasive cancer and ductal carcinoma in situ [DCIS] in the breast and axillary nodes) or ypT0/is ypN0 (absence of invasive cancer in the breast and axillary nodes, irrespective of DCIS) [9,10,11]. As a result, it has been accepted as a valuable prognostic factor for BC patients [12,13,14]. However, only a small fraction of patients who receive NAC will achieve pCR (10–30%) [15,16]. Consequently, predicting pCR before NAC administration is of utmost importance to appropriately stratify patients and to avoid unnecessary chemotherapy toxicity in non-responders, while paving the way for a more personalised treatment strategy.

Among the imaging techniques for BC, magnetic resonance imaging (MRI) has been proven to be the most accurate for measuring treatment response based on the change of tumour size or volume [17]. Several studies have reported the utility of different imaging biomarkers derived from dynamic contrast enhanced (DCE)-MRI in the prediction of early response [18,19,20], while others have pointed out to the apparent diffusion coefficient (ADC) measured using diffusion weighted imaging (DWI) as a surrogate biomarker for both diagnosis and response assessment [21,22,23]. It also seems clear that the combination of DCE-MR imaging with DWI performs better than either method alone in predicting treatment response to NAC [24,25]. In addition, in recent years, mounting evidence suggests that radiomics might become a useful method to assess pCR to NAC in patients with BC [26,27]. Radiomics is a high-throughput quantitative imaging analysis method which extracts a large number of features from medical images [28]. These features are hypothesised to capture underlying information related to the structural heterogeneity and habitats of the entire breast tumour [29,30], allowing the establishment of correlations with clinical or biological endpoints and the building of predictive models by using machine learning tools [31]. However, despite some promising results having been obtained with both MRI-derived parameters and with radiomics, further research is needed to identify robust imaging biomarkers with reduced interobserver variability that allow to predict treatment response to NAC effectively.

The objective of this study was to investigate whether imaging features (perfusion/diffusion imaging biomarkers and radiomic features) extracted from pre-treatment multiparametric (mp)MRI images could non-invasively predict, alone or in combination with clinical variables and by applying machine learning tools, pCR to NAC in patients with BC.

## 2. Materials and Methods

### 2.1. Study Design and Patient Population

This was a retrospective single-centre observational study conducted in accordance with the Declaration of Helsinki and approved by the Institutional Review Board of ethics committee from Castellón Provincial Hospital (Castellón de la Plana, Spain).

Patients with biopsy-proven BC who underwent NAC with or without monoclonal antibody treatment followed by surgery between 1 January 2018 and 31 December 2019 were selected if the following inclusion criteria were met: (a) presenting stage II-III BC; (b) having received NAC with no prior therapy; (c) available pre-treatment standard-of-care breast mpMRIs. Exclusion criteria included incomplete or imaging artifacts at mpMRI examination.

### 2.2. Definition of Pathological Complete Response

The prognostic impact of pCR depends on its definition, which has not yet been standardised [32]. In this study, pCR was defined as the absence of residual invasive carcinoma in the complete resected breast specimen on haematoxylin and eosin evaluation and in all sampled regional lymph nodes following the completion of NAC (pT0 or ypTis and ypN0), although the presence of residual ductal carcinoma in situ was accepted. This definition has been widely used in the literature, as in the studies conducted by the Collaborative Trials in Neoadjuvant Breast Cancer (CTNeoBC) [9] and the MD Anderson [33]. Additionally, it has demonstrated to best discriminate between patients with favourable and unfavourable outcomes [32,34].

In this study, patients showing pCR will be also referred as “responders” opposite to those not showing pCR who will be referred as “non-responders”.

### 2.3. MRI Acquisition

The MRI protocol included multi-slice T1- and T2-weighted fast spin–echo sequences in axial orientation, short time inversion recovery (STIR) sequences, DWI sequences, and DCE sequences. Images were acquired in a Siemens 1.5T MRI unit (Magnetom Avanto fit, Siemens Healthineers, Erlangen, Germany) with the patient lying in a prone position and by using a dedicated breast radiofrequency coil with 18-channel (Breast 18-type).

For DCE series, high-spatial resolution three-dimensional T1-weighted imaging was performed by using a 3D gradient-echo sequence (TE = 2.5 ms, TR = 5.9 ms, flip angle = 25°, isotropic voxel size of 1.2 mm, field of view of 320 mm). A total of six dynamics were acquired with a temporal resolution of 40 s. Total DCE sequence duration was of 7 min 42 s, including preparation pulses. The contrast media used was Gadovist^®^ (Gadobutrol, 0.1 mmol/mL, Bayer AG, Berlin, Germany), administered by the antecubital vein through automatic injector at a dose of 0.1 mmol/Kg with a flow rate of 3 mL/s, followed by 25 mL of physiological saline (OptiStar Elite^®^ Mallinckrodt Pharmaceuticals, Liebel-Flarsheim Company, Cincinnati, OH, USA). Contrast injection was started 20 s after the end of the first dynamic. DWI images were acquired by an echo–planar imaging sequence (TE = 84 ms, TR = 6900 ms, pixel size of 1.9 mm, slice thickness of 5 mm, b-values of 0, 150, 400, and 1000 s^2^/mm).

### 2.4. Lesion Segmentation

Three-dimensional delineation of the tumour was carried out on pre-treatment DCE sequences after digital subtraction by a radiologist from a Breast Unit with more than 10 years of experience.

### 2.5. Imaging Feature Analysis

The segmented tumoral regions were characterised by diffusion and perfusion parameters extracted from DWI and DCE sequences, that provided information about tumour cellularity and vascularisation, respectively. The following imaging biomarkers were extracted:

- DCE:
  o Initial Area under the Curve at 60 seconds (iAUC60) [mM]: area under the concentration curve increment 60 s after the start of contrast administration;
  o Initial slope: represents the initial ascending slope of the concentration curve once the contrast begins to enter the analysed region;
  o Peak [mM]: maximum value of the concentration curve;
  o Time-to-peak (TTP) [s]: time to reach the maximum value of the curve.
- DWI:
  o ADC [mm^2^/s]: Diffusion was quantified using a Gaussian mono exponential diffusion model to obtain;
  o Diffusion Coefficient (D) [mm^2^/s]: Pure diffusion coefficient derived from bi-exponential model of intra-voxel incoherent motions (IVIM);
  o Perfusion Coefficient (D*) [mm^2^/s]: Fast diffusion coefficient derived from bi-exponential model of intra-voxel incoherent motions (IVIM);
  o Vascular fraction (f) [%]: Percentage of the voxel diffusion signal corresponding to fast diffusion.

Since the analysis of diffusion and perfusion biomarkers was performed by a voxel-by-voxel approach in the tumoral region, different statistic metrics were calculated for each of the biomarkers (mean, median, standard deviation, 25th percentile, and 75th percentile).

Subsequently, radiomics features were extracted from tumour region on the ADC and iAUC60 maps to characterise lesion heterogeneity. First-order and second-order features were extracted from the image histogram and by the use of different second-order transformations, consisting of gray-level co-occurrence matrix (GLCM), gray-level run length matrix (GLRLM), gray-level size-zone matrix (GLSZM), or neighbourhood gray-tone difference matrix (NGTDM), as can be found in the paper of the Image Biomarker Standardisation Initiative (IBSI) [35].

The results were analysed in two ways. First, statistical tests for each parameter were carried out to explore whether imaging features (DCE-MRI and DWI-MRI quantitative imaging biomarkers and radiomic features) exhibited statistically significant differences when comparing patients with pCR versus non-responders. In a second step, we explored whether imaging features were sufficient to produce a gain in pCR prediction when combined with clinical data and compared to clinical or imaging data alone, the ultimate goal of this work. To this aim, we trained different machine learning models.

### 2.6. Predictive Model

The objective of the predictive model was to anticipate pCR to NAC based on the analysis of the data obtained from their pre-treatment MRI sequences. This model was based on an artificial intelligence classifier; a machine learning algorithm that automatically orders or categorises data into one or more of a set of “classes”. A process of data curation followed by a homogenisation of the groups was previously required in order to avoid unbalanced categories. This was followed by a two-step feature selection process, in which reproducible, informative, and non-redundant variables were selected. Thus, in a first step, variables with extremely low variance values (≤0.1) were eliminated. In a second step, for variables exhibiting correlation > 0.8, only those with the highest variance were kept.

The following machine learning classifiers were trained and validated:

- K-Nearest Neighbour (K-NN): stores all training data and classifies a new data point according to the class of the majority of its k nearest neighbours in the given dataset. To obtain the nearest neighbours for each data, K-NN uses a measure to compute the distance between pairs of data items [36];
- Decision Tree (DT): organises the knowledge extracted from data in a recursive hierarchical structure composed of nodes and branches. Each internal node represents an attribute and is associated to a test relevant for data classification. Leaf nodes of the tree correspond to classes. Branches represent each of the possible results of the applied tests. A new example can be classified following the nodes and branches accordingly until a leaf node is reached [36];
- Random Forest (RF): a method consisting of combinations of tree predictors. Each tree votes for its preferred class and the most voted class gives the final prediction [36];
- Adaptive Boosting (AdaBoost): an ensemble learning method in which a number of weak learners are combined together to form a strong learner. This method focuses on training upon misclassified observations. It alters the distribution of the training dataset to increase weights on sample observations that are difficult to classify [37];
- Gradient Boosting (GBoost): an ensemble learning method in which a number of weak learners are combined together to form a strong learner. This approach trains learners based upon minimising the loss function of a learner (i.e., training on the residuals of the model) [38];
- Gaussian Naïve Bayes (GNB): probabilistic classifier based on the Bayes theorem for conditional probabilities. It builds a function, to be optimised, using a narrow (naïve) assumption that all attributes in a dataset are independent. It follows Gaussian normal distribution and supports continuous data [36];
- Linear Discriminant Analysis (LDA): a common technique used for dimensionality reduction and classification. LDA provides class separability by drawing a decision region between the different classes. LDA tries to maximize the ratio of the between-class variance and the within-class variance. LDA assumes the feature covariance matrices of both classes are the same [39];
- Quadratic Discriminant Analysis (QDA): a generative model that uses a quadratic decision surface to separate measurements of two or more classes of objects or events. It is a variant of the LDA [39];
- Multi-Layer Perceptron (MLP): a neural network algorithm that learns the relationships between linear and non-linear data. It consists of three different layers in which neurons are trained with the back propagation learning algorithm [40];
- Logistic Regression (LR): statistical models in which a logistic curve is fitted to the dataset, modelling the probability of occurrence of a class. The first step in LR consists of building a logit variable, containing the natural log of the odds of the class occurring or not. A maximum likelihood estimation algorithm is then applied to estimate the probabilities [36].

The leave-one-out cross-validation method was applied to train classifiers and test their performance. This procedure consists of selecting all cases but one to train the algorithm and then, using the one left out for validations. This process was repeated *n* times equal to the total number of cases, leaving out a different case to test on each time. By the end, each training example was left out as a test example once.

To evaluate whether imaging variables produced a gain in pCR prediction compared to clinical data, three different machine learning models were trained with: (1) imaging features (DCE-MRI and DWI-MRI imaging biomarkers + mpMRI-derived radiomic features); (2) clinical data (age at diagnosis (under or over 40 years), menopausal status (premenopausal or not), TNM clinical stage at diagnosis (stage II or stage III), and histological grade in the diagnostic biopsy measured with the Nottingham Histologic Score (grade III or grade I–II)); (3) imaging features + clinical data. Confusion matrices and different metrics (accuracy, sensitivity, specificity, and error rate) were obtained to evaluate model performance.

### 2.7. Statistical Analysis

All statistical analyses were performed in SPSS (IBM Corp., Armonk, NY, USA). A Student’s t-test was used to find possible differences in the imaging features extracted from pre-treatment MRIs between patients showing pCR and non-responders. A *p*-value less than 0.05 was considered statistically significant.

## 3. Results

### 3.1. Clinical Characteristics

Out of the 83 patients diagnosed with stage II–III BC treated with NAC followed by surgery during the study period, only 58 met the inclusion criteria and were ultimately included. The median (range) age of the patients was 52 years (45–58 years). Main patient characteristics are summarised in Table 1.

At diagnosis, 40% women were premenopausal and 68% had operable BC not amenable to conservative surgery. Most patients (32%) presented clinical TNM stage IIA BC. Invasive ductal carcinoma was the most frequent histological type (90%). Regarding the molecular subtype, luminal tumours were more prevalent (42%), followed by HER2+ (40%) and triple-negative tumours (18%). The median ki67 expression was 30% (range = 5–80%).

With respect to the NAC regimen administered, the majority of patients (60%) received AC (adriamycin 60 mg/m^2^, cyclophosphamide 600 mg/m^2^) once every 14 days for four cycles followed by paclitaxel 80 mg/m^2^ weekly for 12 cycles). The remaining 40% received other NAC regimens with trastuzumab and/or pertuzumab. The dose adjustment of the NAC treatment was required in 32% of the patients. After the treatment, pCR was achieved in 21% of the patients (Table 2).

### 3.2. Feature Analysis

A total of 251 imaging characteristics were extracted from pre-treatment mpMRI scans. A first exploratory analysis was conducted in the whole sample population (*N* = 58) in order to evaluate putative differences between responders and non-responders. Out of the 251 (Supplementary Table S1), statistically significant differences were found in four imaging features (Table 3).

**Figure 1:**
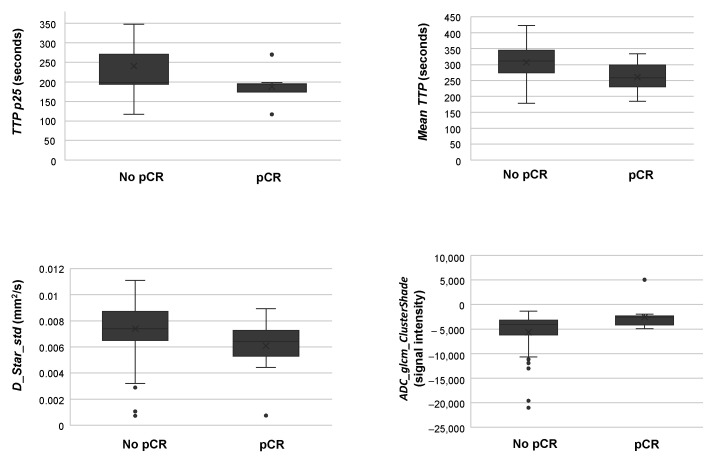
Box-and-whisker plots comparing imaging feature values between patients showing pathological complete response and non-responders.

These variables included one diffusion parameter (the standard deviation of perfusion-related diffusion coefficient, henceforth *D_star_std*), one perfusion parameter (the 25th percentile and the mean of time-to-peak (TTP), henceforth *TTP p25* and *mean TTP*, respectively) and one radiomic feature extracted from DWI-MRI images (Cluster Shade, henceforth *ADC_glcm_ClusterShade*). However, as observed in Figure 1, only *TTP p25*, whose mean value was significantly lower in patients showing pCR and retained a high discriminative ability, being able to differentiate responders from non-responders in 75% of the cases. In line with *TTP p25* results, *mean TTP* was also significantly lower in patients with pCR. On the other hand, *D_star_std* decreased, while *ADC_glcm_ClusterShade* increased in responders compared to non-responders (Table 3 and Figure 1).

Only statistically significant results are presented (*p* < 0.05). “ADC” prefix in *ADC_glcm_ClusterShade* indicates the type of sequence (diffusion) that textural feature was extracted from. *D_star_std* = standard deviation of perfusion-related diffusion coefficient; pCR = pathological complete response; TTP = time-to-peak; *TTP p25* = 25th percentile of time-to-peak.

### 3.3. Predictive Model

**Figure 2:**
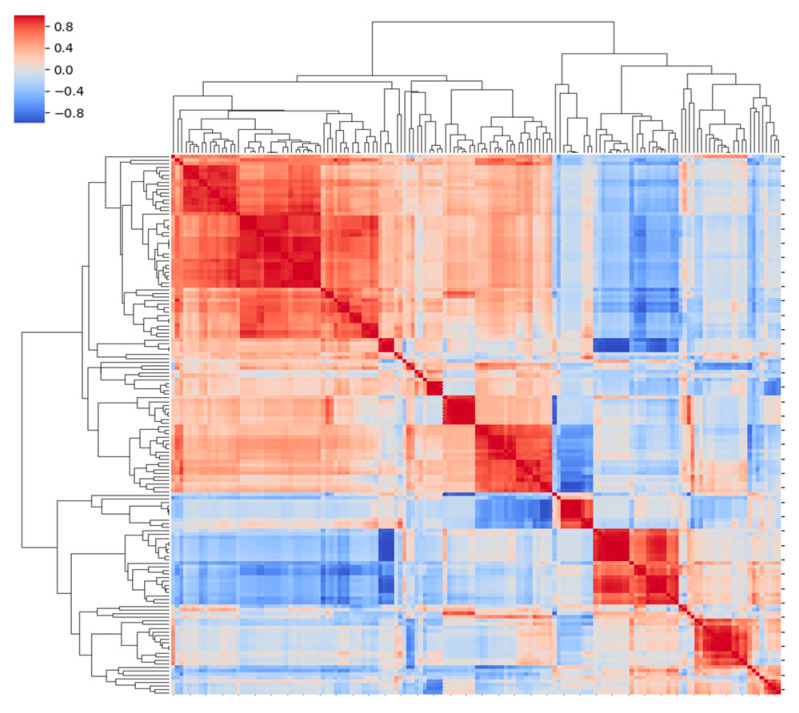
Correlation matrix representing selected imaging features. Colour scale on the left side indicates degree of correlation (from −1 to 1 and from blue to red).

As explained in methodology Section 2.6, an initial data processing was required to build the predictive models. Firstly, since our database included only 12 patients showing pCR, and to avoid overfitting models towards the predominant class, data were balanced by randomly selecting 12 non-responder cases for our analysis. As a result, 24 patients (12 achieving pCR and 12 not achieving pCR) were included to train models. Secondly, through a two-step feature selection process, highly correlated variables and those with low variance values were eliminated. As a result, 38 imaging features were finally selected (Figure 2).

Three different prediction models were developed based on imaging parameters (DCE-MRI and DWI-MRI imaging biomarkers + radiomic features), clinical variables, and the combination of both. Additionally, for each of the models, 10 different machine learning classifiers were tested.

**Figure 3:**
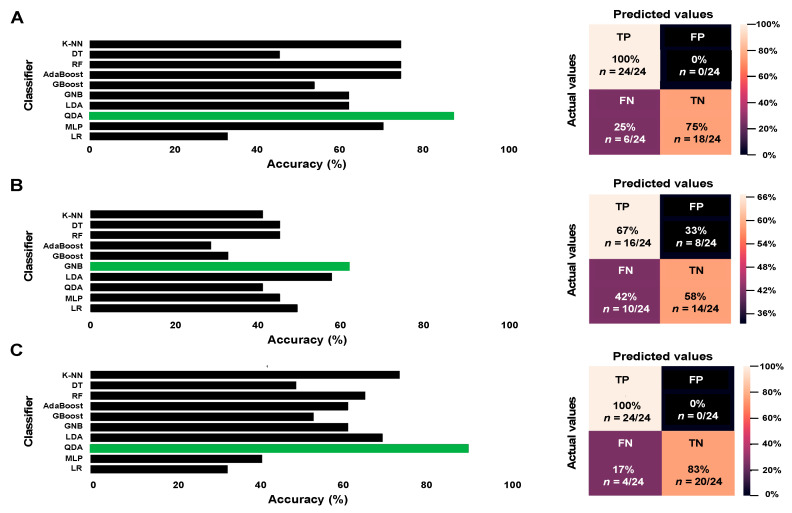
Accuracy values (**left**) for each of the tested classifiers and confusion matrices (**right**) corresponding to models trained with (**A**) imaging features (diffusion/perfusion MRI parameters + radiomic features), (**B**) clinical variables, and (**C**) imaging data + clinical variables. Classifiers achieving the highest accuracy values for each model are highlighted in green. AdaBoost = Adaptive Boosting; DT = Decision Tree; FN = false negative; FP = false positive; GBoost = Gradient Boosting; GNB = Gaussian Naive Bayes; K-NN = K-Nearest Neighbour; LDA = Linear Discriminant Analysis; LG = Logistic Regression; MLP = Multi-Layer Perceptron; QDA = Quadratic Discriminant Analysis; TN = true negative; TP = true positive.

Our results demonstrate that, when only imaging parameters were used, QDA was the classifier yielding the highest accuracy (87.5%) (Figure 3A). In the case of models trained with clinical variables only, GNB yielded the best results, with 62.5% accuracy (Figure 3B). Finally, QDA was also the best classifier in terms of accuracy when both imaging and clinical variables were considered (91.5%) (Figure 3C).

Confusion matrices also confirmed clinical + imaging feature model as the one providing the lowest rate of false negative results (17% vs. 25% in the model trained with imaging features and 42% in the model trained with clinical variables) with no false positive cases (Figure 3).

Finally, the performance of the models according to the input data was evaluated. As shown in Table 4, higher specificity, sensitivity, and accuracy, as well as lower error rates were achieved when imaging data and clinical variables were included in the predictive model.

## 4. Discussion

Response to NAC is one of the most powerful surrogate markers to predict BC prognosis [9], as pCR has been associated with improved EFS and OS [32,41]. Our study was designed to investigate the potential of a combined set of classic perfusion/diffusion MRI biomarkers and radiomic features (referred as imaging data) obtained from pre-treatment images for the prediction of pCR. Our results evidence that incorporating both imaging data and clinical factors into the model facilitates the non-invasive prediction of pCR. This model performed better than those built with only imaging or clinical data, with high accuracy (91.5%) and low error rate (8.5%), providing an effective tool for clinical decision-making.

Several studies have focused on building machine learning models to predict pCR based on imaging features alone or in combination with clinical variables [27,42,43,44,45,46,47,48]. The methodology used in these studies was highly variable, with some of them focused on diffusion/perfusion MRI parameters solely [44,45] and some others exclusively focused on radiomic features extracted from either DCE-MRI sequences [27,42,43] or mpMRI [47,48]. Despite this variability, overall, machine learning models showed good performance in predicting pCR. Additionally, and as confirmed by a meta-analysis recently published [49], pCR prediction by radiomics was more precise when clinical information was included in the model. In our study, we followed a broad approach by firstly, extracting radiomic features from mpMRI without neglecting the importance of diffusion/perfusion MRI parameters that were also evaluated, and secondly, by including clinical variables in our predictive model. Our results demonstrate that the combination of diffusion/perfusion MRI biomarkers and radiomic features along with clinical variables was able to predict pCR with high accuracy, confirming the need for complete prediction models based on a wide range of both imaging and clinical data.

Our statistical analysis revealed significant differences in four distinct variables when comparing patients showing pCR vs. non-responders, including one diffusion parameter, one perfusion parameter and one radiomic feature. Among those, the perfusion parameter *TTP p25* was particularly interesting as it was able to discriminate both groups in 75% of the cases. As for *mean TTP*, *TTP p25* was significantly shorter in patients achieving pCR. TTP is a semi-quantitative DCE-MRI-derived parameter that represents the time in which the contrast agent reaches the peak volume [50], proving useful information about how fast the contrast is delivered, and consequently, about tumour vascularity. Indeed, in BC it has been reported that an initial rapid and delayed washout pattern with short TTP may be suggestive of a highly cellular tumour with a relatively small amount of interstitium, where the tumour supplying the vessel is highly permeable [50,51]. Thus, based on our results, we hypothesise that patients showing pCR would be those initially presenting hypervascular tumours, with more accessible vessels to receive chemotherapy agents and consequently, to facilitate therapeutic response. Similar vascular changes and their relation to NAC response have been previously suggested in BC by other authors [52]. Although DWI-MRI parameters may also provide useful information on tumour vascularisation [53], our results only revealed differences between responders and non-responders in one diffusion parameter, *D_star_std*, a variable representing the standard deviation of Dstar and consequently, difficult to interpret. However, it should be noted that, despite this coefficient representing incoherent microcirculation [54], which has been considered proportional to average blood velocity and capillary segment length [55], it seems not to be reliable, showing poor reproducibility [54,56,57]. Additionally, Dstar and other diffusion information only show moderate correlation with pharmacokinetic parameters obtained from DCE-MRI in breasts [58]; an additional reason why vascular changes in patients with pCR could not be reflected in changes in DWI parameters in our study.

Finally, our statistical analysis also showed a significant increase in *Adc_glcm_ClusterShade*, a radiomic feature representing the skewness and uniformity of the GLCM. GLCM is a statistical matrix that has been extensively used in texture analysis [59], a methodology that provides an objective, quantitative assessment of tumour heterogeneity [60]. It has been postulated that texture analysis could provide physicians with additional information to increase the accuracy of prediction of an individual response before NAC is started [61,62]. In our series, more negative *Adc_glcm_ClusterShade* values were obtained in non-responders. Although not significant, similar results were obtained in a study assessing chemotherapy response in BC through texture analysis, in which the authors concluded that a lack of response, and consequently a poorer prognosis, was related to a higher tumour heterogeneity [63].

In summary, our statistical analysis results might suggest that those patients presenting with highly vascularised homogeneous tumours would be more prone to achieve pCR, and therefore, would be ideal candidates to receive NAC.

However, this study has also some limitations. Firstly, it was a single-centre study and its application to data/patients from other institutions should be further explored. Secondly, given the need for a balanced dataset, the final sample used for machine learning models was small. Finally, we are aware that the presence of different tumour subtypes may be affecting the outcomes. Regrettably, because of the small sample size, we were unable to run independent analyses for each of the different subtypes. Additionally, it is worth mentioning that this information, as well as some other relevant clinical information, such as the histology type, was not available when predictive models were developed and consequently, we were unable to include it in the models. Despite its unquestionable relevance in assessing patients’ pCR, we would like to highlight the valuable contribution of the imaging features to the predictive model, which even in combination with the limited clinical information available, were able to improve the performance of models trained only with clinical data or with imaging features. As this is a first proof-of-principle study, we hope to address all these limitations in future studies. Despite them, this work provides valuable insights about pCR prediction in the daily clinical setting. As a result, it contributes to generate real-world evidence, a relevant type of clinical evidence which is drawing ever-increasing attention in the pharmaceutical industry and drug regulatory authorities all over the world.

## 5. Conclusions

In conclusion, a machine learning model, including mpMRI-derived diffusion/perfusion parameters, radiomic features, as well as clinical data obtained in the real-world setting, was able to improve the pre-treatment prediction of pCR to NAC compared to models that only included imaging or clinical data. Pre-treatment differences in specific mpMRI-derived parameters between responders and non-responders might suggest an increased tumour vascularity and decreased heterogeneity in those showing pCR, where *TTP p25* could potentially emerge as a surrogate imaging biomarker for pCR. Further studies are needed to elucidate the predictive value of this parameter, to identify other potential imaging biomarkers, as well as to confirm the added predictive value of imaging features over clinical data in bigger and more complete patient cohorts.

## Figures and Tables

**Table 1:** Patient characteristics at baseline and treatment received.

Characteristic	Patients(*N* = 58) *n* (%)
**Premenopausal**	23 (40)
**Clinical TNM stage**	
IIA	19 (32)
IIB	10 (18)
IIIA	9 (16)
IIIB	13 (22)
IIIC	7 (12)
**Histology**	
Ductal	52 (90)
Others	6 (10)
Associated DISC	20 (34)
**Nottingham Histologic Score (Grade II)**	37 (64)
**Lymphovascular invasion**	18 (30)
**Molecular subtype**	
Luminal A	15 (26)
Luminal B	9 (15)
HER2+	23 (40)
Triple-negative	11 (19)
**Neoadjuvant chemotherapy**	
ddAC + paclitaxel weekly	35 (60)
ddAC + paclitaxel + CBDCA AUC2	8 (14)
NAC with trastuzumab + pertuzumab	19 (32)
Clinical trial with trastuzumab + pertuzumab	4 (7)
**Conservative surgery**	26 (45)
**Negative margins**	57 (99)
**Residual tumour**	
No	17 (29)
Yes	41 (71)
I	31 (54)
II	5 (8)
III	5 (8)
**Systemic adjuvant treatment**	
Adjuvant chemotherapy	10 (18)
Adjuvant hormone therapy	34 (59)
Adjuvant trastuzumab	23 (40)
**Adjuvant radiation therapy**	52 (90)

AC = doxorubicin/cyclophosphamide; AUC = area under the curve; CBDCA = carboplatin; dd = dose-dense.

**Table 2:** Response assessment after neoadjuvant treatment.

Type of Response	*n* (%)
**Clinical response**	
SD	15 (26)
PR	24 (42)
CR	19 (32)
**Radiological response**	
SD	7 (12)
PR	25 (44)
CR	25 (44)
**pCR (ypT0/is ypN0)**	
Yes	12 (21)
No	46 (79)

CR = complete response; HER2 = human epidermal growth factor receptor 2; PR = partial response; pCR = pathological complete response; SD = stable disease.

**Table 3:** Results of the imaging feature analysis according to the presence or absence of pathological complete response. Only statistically significant results are presented (*p* < 0.05). “ADC” prefix in *ADC_glcm_ClusterShade* indicates the type of sequence (diffusion) that textural feature was extracted from.

Imaging Feature	Mean (SD)	*p*-Value
***TTP p25 (s)***		
No pCR	237.67 (79.20)	0.004
pCR	187.47 (51.27)	
***mean*** ***TTP (s)***		
No pCR	302.40 (56.67)	0.026
pCR	260.52 (46.61)	
***D_star_std (mm^2^/s)***		
No pCR	0.007 (0.002)	0.012
pCR	0.006 (0.002)	
***ADC_glcm_ClusterShade*** ***(signal intensity; absolute value)***		
No pCR	−5140.14 (3644.81)	0.035
pCR	−2542.84 (2607.65)	

D_star_std = standard deviation of perfusion-related diffusion coefficient; pCR = pathological complete response; TTP = time-to-peak; TTP p25 = 25th percentile of time-to-peak; SD, standard deviation.

**Table 4:** Performance metrics of the different predictive models. Only metrics for the classifier providing the best results for each of the models are detailed.

	Predictive Models	Imaging Data QDA Classifier	Clinical Data GNB Classifier	Imaging + Clinical DataQDA Classifier
Performance				
Sensitivity		100%	63%	100%
Specificity		80%	61.5%	85.5%
Error rate		12.5%	37.5%	8.5%
Accuracy		87.5%	62.5%	91.5%

GNB = Gaussian Naive Bayes; QDA = Quadratic Discriminant Analysis.

## Data Availability

The data presented in this study are available on request from the corresponding author.

## Supplementary Materials

The following supporting information can be downloaded at: https://www.mdpi.com/article/10.3390/cancers14143508/s1, Table S1. Imaging feature analysis results. P-values from t-Student test results are provided. “ADC” and “iAUC” prefixes before textural features indicate the type of sequence (diffusion and perfusion. respectively) those parameters were extracted from.
