# Supplementary material for: Machine Learning Models and Multiparametric Magnetic Resonance Imaging for the Prediction of Pathologic Response to Neoadjuvant Chemotherapy in Breast Cancer
Source: Cancers (Basel). 2022 Jul 19;14(14):3508. doi: 10.3390/cancers14143508 (PMC9317428; doi:10.3390/cancers14143508)
Supplement: Supplementary file 1 [file cancers-14-03508-s001.zip › cancers-1758087-supplementary.pdf]

**Supplementary Table S1**. Imaging feature analysis results. P-values from t-Student test results are provided. “ADC” and “iAUC” prefixes before textural features indicate the type of sequence (diffusion and perfusion. respectively) those parameters were extracted from.

| | **Imaging feature** | ***p*-value** |
|---|---|---|
| 1 | *iAUC_shape_MeshVolume* | 0.865 |
| 2 | *D_mean* | 0.622 |
| 3 | *D_std* | 0.810 |
| 4 | *D_median* | 0.705 |
| 5 | *D p25* | 0.798 |
| 6 | *D p75* | 0.425 |
| 7 | *D_star_mean* | 0.867 |
| 8 | *D_star_std* | 0.012 |
| 9 | *D_star_median* | 0.567 |
| 10 | *D_star p25* | 0.372 |
| 11 | *D_star p75* | 0.293 |
| 12 | *f_mean* | 0.697 |
| 13 | *f_std* | 0.956 |
| 14 | *f_median* | 0.926 |
| 15 | *f p25* | 0.727 |
| 16 | *f p75* | 0.635 |
| 17 | *ADC_mean* | 0.698 |
| 18 | *ADC_std* | 0.935 |
| 19 | *ADC_median* | 0.694 |
| 20 | *ADC p25* | 0.923 |
| 21 | *ADC p75* | 0.423 |
| 22 | *iAUC_mean* | 0.162 |
| 23 | *iAUC_std* | 0.609 |
| 24 | *iAUC_median* | 0.121 |
| 25 | *iAUC p25* | 0.057 |
| 26 | *iAUC p75* | 0.200 |
| 27 | *IS_mean* | 0.538 |
| 28 | *IS_std* | 0.077 |
| 29 | *IS_median* | 0.915 |
| 30 | *IS p25* | 0.481 |
| 31 | *IS p75* | 0.720 |
| 32 | *Peak_mean* | 0.548 |
| 33 | *Peak_std* | 0.079 |
| 34 | *Peak_median* | 0.987 |
| 35 | *Peak p25* | 0.610 |
| 36 | *Peak p75* | 0.634 |
| 37 | *TTP_mean* | 0.026 |
| 38 | *TTP_std* | 0.462 |

| | | |
|---|---|---|
| 39 | *TTP_median* | 0.064 |
| 40 | *TTP p25* | 0.004 |
| 41 | *TTP p75* | 0.116 |
| 42 | *ADC_shape_Elongation* | 0.587 |
| 43 | *ADC_shape_MajorAxisLength* | 0.436 |
| 44 | *ADC_shape_MinorAxisLength* | 0.680 |
| 45 | *ADC_shape_Sphericity* | 0.929 |
| 46 | *ADC_shape_Flatness* | 0.697 |
| 47 | *ADC_shape_LeastAxisLength* | 0.742 |
| 48 | *ADC_shape_Maximum2DDiameterColumn* | 0.432 |
| 49 | *ADC_shape_Maximum2DDiameterRow* | 0.872 |
| 50 | *ADC_shape_Maximum2DDiameterSlice* | 0.699 |
| 51 | *ADC_shape_Maximum3DDiameter* | 0.603 |
| 52 | *ADC_shape_MeshVolume* | 0.728 |
| 53 | *ADC_shape_SurfaceArea* | 0.891 |
| 54 | *ADC_shape_SurfaceVolumeRatio* | 0.710 |
| 55 | *ADC_shape_VoxelVolume* | 0.732 |
| 56 | *ADC_firstorder_10Percentile* | 0.496 |
| 57 | *ADC_firstorder_90Percentile* | 0.579 |
| 58 | *ADC_firstorder_Energy* | 0.737 |
| 59 | *ADC_firstorder_Entropy* | 0.616 |
| 60 | *ADC_firstorder_InterquartileRange* | 0.369 |
| 61 | *ADC_firstorder_Kurtosis* | 0.564 |
| 62 | *ADC_firstorder_Maximum* | 0.249 |
| 63 | *ADC_firstorder_MeanAbsoluteDeviation* | 0.528 |
| 64 | *ADC_firstorder_mean* | 0.414 |
| 65 | *ADC_firstorder_median* | 0.393 |
| 66 | *ADC_firstorder_minimum* | 0.962 |
| 67 | *ADC_firstorder_range* | 0.831 |
| 68 | *ADC_firstorder_RobustMeanAbsoluteDeviation* | 0.454 |
| 69 | *ADC_firstorder_RootMeanSquared* | 0.411 |
| 70 | *ADC_firstorder_Skewness* | 0.381 |
| 71 | *ADC_firstorder_TotalEnergy* | 0.737 |
| 72 | *ADC_firstorder_Uniformity* | 0.871 |
| 73 | *ADC_firstorder_Variance* | 0.461 |
| 74 | *ADC_glcm_Autocorrelation* | 0.504 |
| 75 | *ADC_glcm_JointAverage* | 0.514 |
| 76 | *ADC_glcm_ClusterProminence* | 0.474 |
| 77 | *ADC_glcm_ClusterShade* | 0.035 |
| 78 | *ADC_glcm_ClusterTendency* | 0.422 |
| 79 | *ADC_glcm_Contrast* | 0.392 |
| 80 | *ADC_glcm_Correlation* | 0.811 |
| 81 | *ADC_glcm_DifferenceAverage* | 0.465 |

| | | |
|---|---|---|
| 82 | *ADC_glcm_DifferenceEntropy* | 0.526 |
| 83 | *ADC_glcm_DifferenceVariance* | 0.623 |
| 84 | *ADC_glcm_JointEnergy* | 0.932 |
| 85 | *ADC_glcm_JointEntropy* | 0.865 |
| 86 | *ADC_glcm_Imc1* | 0.641 |
| 87 | *ADC_glcm_Imc2* | 0.917 |
| 88 | *ADC_glcm_Idm* | 0.567 |
| 89 | *ADC_glcm_Idmn* | 0.886 |
| 90 | *ADC_glcm_Id* | 0.553 |
| 91 | *ADC_glcm_Idn* | 0.672 |
| 92 | *ADC_glcm_InverseVariance* | 0.529 |
| 93 | *ADC_glcm_MaximumProbability* | 0.660 |
| 94 | *ADC_glcm_SumEntropy* | 0.518 |
| 95 | *ADC_glcm_SumSquares* | 0.436 |
| 96 | *ADC_glrlm_GrayLevelNonUniformity* | 0.753 |
| 97 | *ADC_glrlm_GrayLevelNonUniformityNormalized* | 0.860 |
| 98 | *ADC_glrlm_GrayLevelVariance* | 0.481 |
| 99 | *ADC_glrlm_HighGrayLevelRunEmphasis* | 0.563 |
| 100 | *ADC_glrlm_LongRunEmphasis* | 0.548 |
| 101 | *ADC_glrlm_LongRunHighGrayLevelEmphasis* | 0.401 |
| 102 | *ADC_glrlm_LongRunLowGrayLevelEmphasis* | 0.885 |
| 103 | *ADC_glrlm_LowGrayLevelRunEmphasis* | 0.874 |
| 104 | *ADC_glrlm_RunEntropy* | 0.794 |
| 105 | *ADC_glrlm_RunLengthNonUniformity* | 0.801 |
| 106 | *ADC_glrlm_RunLengthNonUniformityNormalized* | 0.603 |
| 107 | *ADC_glrlm_RunPercentage* | 0.606 |
| 108 | *ADC_glrlm_RunVariance* | 0.488 |
| 109 | *ADC_glrlm_ShortRunEmphasis* | 0.645 |
| 110 | *ADC_glrlm_ShortRunHighGrayLevelEmphasis* | 0.623 |
| 111 | *ADC_glrlm_ShortRunLowGrayLevelEmphasis* | 0.873 |
| 112 | *ADC_glszm_GrayLevelNonUniformity* | 0.847 |
| 113 | *ADC_glszm_GrayLevelNonUniformityNormalized* | 0.651 |
| 114 | *ADC_glszm_GrayLevelVariance* | 0.526 |
| 115 | *ADC_glszm_HighGrayLevelZoneEmphasis* | 0.604 |
| 116 | *ADC_glszm_LargeAreaEmphasis* | 0.876 |
| 117 | *ADC_glszm_LargeAreaHighGrayLevelEmphasis* | 0.739 |
| 118 | *ADC_glszm_LargeAreaLowGrayLevelEmphasis* | 0.986 |
| 119 | *ADC_glszm_LowGrayLevelZoneEmphasis* | 0.926 |
| 120 | *ADC_glszm_SizeZoneNonUniformity* | 0.763 |
| 121 | *ADC_glszm_SizeZoneNonUniformityNormalized* | 0.487 |
| 122 | *ADC_glszm_SmallAreaEmphasis* | 0.484 |
| 123 | *ADC_glszm_SmallAreaHighGrayLevelEmphasis* | 0.666 |
| 124 | *ADC_glszm_SmallAreaLowGrayLevelEmphasis* | 0.923 |

| | | |
|---|---|---|
| 125 | *ADC_glszm_ZoneEntropy* | 0.864 |
| 126 | *ADC_glszm_ZonePercentage* | 0.531 |
| 127 | *ADC_glszm_ZoneVariance* | 0.877 |
| 128 | *ADC_gldm_DependenceEntropy* | 0.830 |
| 129 | *ADC_gldm_DependenceNonUniformity* | 0.787 |
| 130 | *ADC_gldm_DependenceNonUniformityNormalized* | 0.439 |
| 131 | *ADC_gldm_DependenceVariance* | 0.563 |
| 132 | *ADC_gldm_GrayLevelNonUniformity* | 0.741 |
| 133 | *ADC_gldm_GrayLevelVariance* | 0.460 |
| 134 | *ADC_gldm_HighGrayLevelEmphasis* | 0.548 |
| 135 | *ADC_gldm_LargeDependenceEmphasis* | 0.648 |
| 136 | *ADC_gldm_LargeDependenceHighGrayLevelEmphasis* | 0.409 |
| 137 | *ADC_gldm_LargeDependenceLowGrayLevelEmphasis* | 0.869 |
| 138 | *ADC_gldm_LowGrayLevelEmphasis* | 0.853 |
| 139 | *ADC_gldm_SmallDependenceEmphasis* | 0.509 |
| 140 | *ADC_gldm_SmallDependenceHighGrayLevelEmphasis* | 0.977 |
| 141 | *ADC_gldm_SmallDependenceLowGrayLevelEmphasis* | 0.849 |
| 142 | *ADC_ngtdm_Busyness* | 0.902 |
| 143 | *ADC_ngtdm_Coarseness* | 0.436 |
| 144 | *ADC_ngtdm_Complexity* | 0.944 |
| 145 | *ADC_ngtdm_Contrast* | 0.604 |
| 146 | *ADC_ngtdm_Strength* | 0.396 |
| 147 | *iAUC_shape_Elongation* | 0.340 |
| 148 | *iAUC_shape_MajorAxisLength* | 0.601 |
| 149 | *iAUC_shape_MinorAxisLength* | 0.696 |
| 150 | *iAUC_shape_Sphericity* | 0.259 |
| 151 | *iAUC_shape_Flatness* | 0.274 |
| 152 | *iAUC_shape_LeastAxisLength* | 0.982 |
| 153 | *iAUC_shape_Maximum2DDiameterColumn* | 0.419 |
| 154 | *iAUC_shape_Maximum2DDiameterRow* | 0.878 |
| 155 | *iAUC_shape_Maximum2DDiameterSlice* | 0.660 |
| 156 | *iAUC_shape_Maximum3DDiameter* | 0.597 |
| 157 | *iAUC_shape_MeshVolume* | 0.864 |
| 158 | *iAUC_shape_SurfaceArea* | 0.686 |
| 159 | *iAUC_shape_SurfaceVolumeRatio* | 0.696 |
| 160 | *iAUC_shape_VoxelVolume* | 0.898 |
| 161 | *iAUC_firstorder_p10* | 0.768 |
| 162 | *iAUC_firstorder_p90* | 0.824 |
| 163 | *iAUC_firstorder_Energy* | 0.922 |
| 164 | *iAUC_firstorder_Entropy* | 0.328 |
| 165 | *iAUC_firstorder_InterquartileRange* | 0.998 |
| 166 | *iAUC_firstorder_Kurtosis* | 0.777 |
| 167 | *iAUC_firstorder_maximum* | 0.890 |

| | | |
|---|---|---|
| 168 | *iAUC_firstorder_MeanAbsoluteDeviation* | 0.954 |
| 169 | *iAUC_firstorder_mean* | 0.830 |
| 170 | *iAUC_firstorder_median* | 0.899 |
| 171 | *iAUC_firstorder_minimum* | 0.909 |
| 172 | *iAUC_firstorder_range* | 0.973 |
| 173 | *iAUC_firstorder_RobustMeanAbsoluteDeviation* | 0.745 |
| 174 | *iAUC_firstorder_RootMeanSquared* | 0.802 |
| 175 | *iAUC_firstorder_Skewness* | 0.866 |
| 176 | *iAUC_firstorder_TotalEnergy* | 0.922 |
| 177 | *iAUC_firstorder_Uniformity* | 0.747 |
| 178 | *iAUC_firstorder_Variance* | 0.867 |
| 179 | *iAUC_glcm_Autocorrelation* | 0.942 |
| 180 | *iAUC_glcm_JointAverage* | 0.916 |
| 181 | *iAUC_glcm_ClusterProminence* | 0.281 |
| 182 | *iAUC_glcm_ClusterShade* | 0.967 |
| 183 | *iAUC_glcm_ClusterTendency* | 0.559 |
| 184 | *iAUC_glcm_Contrast* | 0.264 |
| 185 | *iAUC_glcm_Correlation* | 0.278 |
| 186 | *iAUC_glcm_DifferenceAverage* | 0.208 |
| 187 | *iAUC_glcm_DifferenceEntropy* | 0.538 |
| 188 | *iAUC_glcm_DifferenceVariance* | 0.719 |
| 189 | *iAUC_glcm_JointEnergy* | 0.983 |
| 190 | *iAUC_glcm_JointEntropy* | 0.598 |
| 191 | *iAUC_glcm_Imc1* | 0.886 |
| 192 | *iAUC_glcm_Imc2* | 0.549 |
| 193 | *iAUC_glcm_Idm* | 0.507 |
| 194 | *iAUC_glcm_Idmn* | 0.335 |
| 195 | *iAUC_glcm_Id* | 0.445 |
| 196 | *iAUC_glcm_Idn* | 0.272 |
| 197 | *iAUC_glcm_InverseVariance* | 0.669 |
| 198 | *iAUC_glcm_MaximumProbability* | 0.686 |
| 199 | *iAUC_glcm_SumEntropy* | 0.706 |
| 200 | *iAUC_glcm_SumSquares* | 0.915 |
| 201 | *iAUC_glrlm_GrayLevelNonUniformity* | 0.853 |
| 202 | *iAUC_glrlm_GrayLevelNonUniformityNormalized* | 0.967 |
| 203 | *iAUC_glrlm_GrayLevelVariance* | 0.707 |
| 204 | *iAUC_glrlm_HighGrayLevelRunEmphasis* | 0.979 |
| 205 | *iAUC_glrlm_LongRunEmphasis* | 0.937 |
| 206 | *iAUC_glrlm_LongRunHighGrayLevelEmphasis* | 0.778 |
| 207 | *iAUC_glrlm_LongRunLowGrayLevelEmphasis* | 0.745 |
| 208 | *iAUC_glrlm_LowGrayLevelRunEmphasis* | 0.882 |
| 209 | *iAUC_glrlm_RunEntropy* | 0.506 |
| 210 | *iAUC_glrlm_RunLengthNonUniformity* | 0.848 |

| | | |
|---|---|---|
| 211 | *iAUC_glrlm_RunLengthNonUniformityNormalized* | 0.441 |
| 212 | *iAUC_glrlm_RunPercentage* | 0.497 |
| 213 | *iAUC_glrlm_RunVariance* | 0.826 |
| 214 | *iAUC_glrlm_ShortRunEmphasis* | 0.588 |
| 215 | *iAUC_glrlm_ShortRunHighGrayLevelEmphasis* | 0.926 |
| 216 | *iAUC_glrlm_ShortRunLowGrayLevelEmphasis* | 0.895 |
| 217 | *iAUC_glszm_GrayLevelNonUniformity* | 0.911 |
| 218 | *iAUC_glszm_GrayLevelNonUniformityNormalized* | 0.740 |
| 219 | *iAUC_glszm_GrayLevelVariance* | 0.784 |
| 220 | *iAUC_glszm_HighGrayLevelZoneEmphasis* | 0.949 |
| 221 | *iAUC_glszm_LargeAreaEmphasis* | 0.869 |
| 222 | *iAUC_glszm_LargeAreaHighGrayLevelEmphasis* | 0.752 |
| 223 | *iAUC_glszm_LargeAreaLowGrayLevelEmphasis* | 0.662 |
| 224 | *iAUC_glszm_LowGrayLevelZoneEmphasis* | 0.794 |
| 225 | *iAUC_glszm_SizeZoneNonUniformity* | 0.820 |
| 226 | *iAUC_glszm_SizeZoneNonUniformityNormalized* | 0.769 |
| 227 | *iAUC_glszm_SmallAreaEmphasis* | 0.855 |
| 228 | *iAUC_glszm_SmallAreaHighGrayLevelEmphasis* | 0.875 |
| 229 | *iAUC_glszm_SmallAreaLowGrayLevelEmphasis* | 0.817 |
| 230 | *iAUC_glszm_ZoneEntropy* | 0.631 |
| 231 | *iAUC_glszm_ZonePercentage* | 0.347 |
| 232 | *iAUC_glszm_ZoneVariance* | 0.885 |
| 233 | *iAUC_gldm_DependenceEntropy* | 0.586 |
| 234 | *iAUC_gldm_DependenceNonUniformity* | 0.901 |
| 235 | *iAUC_gldm_DependenceNonUniformityNormalized* | 0.367 |
| 236 | *iAUC_gldm_DependenceVariance* | 0.358 |
| 237 | *iAUC_gldm_GrayLevelNonUniformity* | 0.791 |
| 238 | *iAUC_gldm_GrayLevelVariance* | 0.868 |
| 239 | *iAUC_gldm_HighGrayLevelEmphasis* | 0.956 |
| 240 | *iAUC_gldm_LargeDependenceEmphasis* | 0.643 |
| 241 | *iAUC_gldm_LargeDependenceHighGrayLevelEmphasis* | 0.767 |
| 242 | *iAUC_gldm_LargeDependenceLowGrayLevelEmphasis* | 0.822 |
| 243 | *iAUC_gldm_LowGrayLevelEmphasis* | 0.889 |
| 244 | *iAUC_gldm_SmallDependenceEmphasis* | 0.339 |
| 245 | *iAUC_gldm_SmallDependenceHighGrayLevelEmphasis* | 0.367 |
| 246 | *iAUC_gldm_SmallDependenceLowGrayLevelEmphasis* | 0.975 |
| 247 | *iAUC_ngtdm_Busyness* | 0.801 |
| 248 | *iAUC_ngtdm_Coarseness* | 0.892 |
| 249 | *iAUC_ngtdm_Complexity* | 0.310 |
| 250 | *iAUC_ngtdm_Contrast* | 0.607 |
| 251 | *iAUC_ngtdm_Strength* | 0.728 |

ADC = apparent diffusion coefficient; D = diffusion coefficient; f = vascular fraction; glcm = gray-level co-occurrence matrix, glrm = gray-level run length matrix; glsm = gray-level size-zone matrix; iAUC = initial area under the contrast agent concentration-time curve; IS = initial slope; ngtdm =

neighbourhood gray-tone difference matrix; p = percentile; std = standard deviation; TTP = time-to-peak;
